# Assessing the Impact of Circulating Tumor DNA (ctDNA) in Patients With Colorectal Cancer: Separating Fact From Fiction

**DOI:** 10.3389/fonc.2018.00297

**Published:** 2018-08-06

**Authors:** Emmanuel Gabriel, Sanjay P. Bagaria

**Affiliations:** Section of Surgical Oncology, Department of Surgery, Mayo Clinic Florida, Jacksonville, FL, United States

**Keywords:** circulating tumor DNA, colorectal cancer, biomarker, cancer diagnosis, treatment

## Abstract

Significant advances and increased awareness have been in made in the field of non-invasive liquid biopsies for cancer, spanning several malignancies from gastrointestinal, pulmonary, and other etiologies. Broadly, the genetic source material for liquid biopsies includes circulating tumor cells, cell-free circulating tumor DNA (ctDNA), or cell-free circulating tumor microRNA (mRNA). In this review, we specifically focus on ctDNA and its current role in colorectal cancer. While there are several commercially available assays that detect ctDNA, the utility of these products is still variable and therefore the clinical applications of ctDNA in the management of patients with cancer has yet to be determined. This is reflected by the recent joint review set forth by the American Society of Clinical Oncology (ASCO) and the College of American Pathologists (CAP), clarifying and somewhat tempering the present role of ctDNA in patients with cancer. This review provides additional detail regarding ctDNA in the limited setting of colorectal cancer. The increasing importance and promise of ctDNA remains an area of active research, and further prospective studies may enhance the clinical utility of ctDNA in the future.

## Introduction

The concept of circulating, cell-free DNA was initially proposed through work by Mandel and Métais in the late 1940s (1, 2). The broad correlation between cell-free DNA and cancer was later observed through work by Leon et al. in the 1970s, where levels of cell-free circulating DNA (though not necessarily tumor specific cell-free DNA) corresponded to the burden of metastatic disease for patients with a variety of tumors, including lymphoma, colorectal, lung, gynecological, breast, and brain tumors (3). The now commonly used term “liquid biopsies” was introduced by Pantel and Alix-Panabières in the late 1980s as a potential means to obtain diagnostic data from the peripheral blood of cancer patients that would have the same function as that derived from tumor specimens (4). The diagnostic source derived from the peripheral blood includes a variety of soluble factors, including cell-free circulating tumor DNA, circulating tumor microRNA (5), proteins and other biomarkers, and intact circulating tumor cells themselves (6). Since then and most significantly with the last decade, research investigating circulating tumor DNA (ctDNA) has expanded rapidly. In fact, a PubMed search using the term “ctDNA” will generate over 3,500 citations on this topic. This number of citations decreases to 403 during a search for “ctDNA and colorectal cancer.”

ctDNA refers to the cell-free DNA released by tumor cells through a variety of proposed mechanisms, including secretion of tumor-associated DNA, necrosis from nonviable tumor cells, or through phagocytosis by tumor infiltrating immune cells (7). Interestingly, the ctDNA fragments themselves may not only represent solid tumor DNA, but also may have the ability to induce oncogenic changes in normal cells that ctDNA encounters, potentially serving as a mechanism for cancer metastasis (8). While these observations have been made only in animal models incorporating human ctDNA, this data suggests a functional role for ctDNA in addition to its more highly studied diagnostic and prognostic roles.

Indeed, there has been much attention recently to ctDNA and its diagnostic/prognostic roles in cancer (9–11). Like all applications for liquid biopsies, ctDNA offers potential advantages over traditional solid tumor biopsies. These include a less invasive means of obtaining diagnostic information through a blood test (as opposed to a percutaneous or open approach to sampling tumor tissue), which can be more frequently and easily repeated with minimal risk to the patient. In addition, the use of ctDNA may result in obtaining a more thorough representation of the tumor heterogeneity that is present within the tumors themselves (12, 13). In contrast, percutaneous tumor biopsies are more limited in sampling heterogeneous areas of the tumor. While a general correlation between high levels of cell-free DNA and cancer has been noted by several groups (2, 3), ctDNA that is specific to known mutations associated with a given malignancy has been targeted as a potential clinical biomarker. In this regard, several studies have demonstrated that ctDNA possesses clinical validity, meaning that it correlates to whether the patient actually has cancer. However, as will be discussed, it remains unclear whether ctDNA possesses clinical utility, meaning the ability of ctDNA to positively impact patient outcomes as a biomarker. In this review, we focus on ctDNA and colorectal cancer, evaluating and its current and potential clinical roles as well as its benefits and limitations (Table 1).

**Table 1:** Summary of studies involving ctDNA and colorectal cancer, presented by topic subheading within the review.

**Result**	**Study(ies)**	**Design**
**TARGETED MUTATIONS IN ctDNA**		
KRAS detection	Ryan et al. (14) Mouliere et al. (12) Thierry et al. (15)	Prospective study of 94 patients showed mutated KRAS ctDNA detection in all stages of CRC Mouse xenograft model of human CRC cell line showed ctDNA serum production and detection Prospective study of 106 patients showed high detection ability of mutated KRAS ctDNA
BRAF detection	Mouliere et al. (12) Thierry et al. (15)	Mouse xenograft model of human CRC cell line showed ctDNA serum production and detection Prospective study of 106 patients showed high detection ability of mutated BRAF ctDNA
APC detection	Wang et al. (16)	Retrospective analysis of 104 patients with CRC showed detection of APC mutated ctDNA
mSEPT9 detection	deVos et al. (17) Grutzmann et al. (18) Toth et al. (19)	Prospective study of 97 patients showing high specificity of mSEPT9 ctDNA with CRC Prospective study of 354 patients showing high specificity of mSEPT9 ctDNA with CRC Prospective study of 60 patients showing high specificity of mSEPT9 ctDNA with CRC but not in benign adenomas
**ctDNA IN EARLY AND LATE COLORECTAL CANCER**		
ctDNA detection associated with worse prognosis in metastatic CRC	Lefebure et al. (28) Bachet et al. (29)	Retrospective analysis of 29 patients with metastatic CRC showed association of ctDNA with worse disease-free survival Prospective study of 425 patients showed correlation between plasma ctDNA and tumor mutations of approximately 70% in patients with liver metastases
ctDNA detection was not associated with worse prognosis in metastatic or locally advanced disease	Strickler et al. (32)	Retrospective analysis of 1,397 patients with mutated EGFR ctDNA
ctDNA in the detection of early CRC disease has not been shown	Lecomte et al. (32) Lin et al. (20)	Retrospective study of 191 patients with stage I-III CRC showed low sensitivity of ctDNA to detect early disease Prospective study of 191 patients with stage I-III CRC showed low sensitivity of ctDNA to detect early disease
ctDNA may be used as a screening tool, but has not been definitively shown	Flamini et al. (41) Mead et al. (40)	Prospective study of 75 patients with known CRC showing elevated ctDNA compared to healthy patients Prospective study of 26 patients showed mutated ctDNA was associated with invasive carcinoma among polypectomies when combined with CEA levels
**ctDNA TO PREDICT PROGNOSIS FOLLOWING SURGERY AND DURING SYSTEMIC TREATMENT**		
ctDNA levels may be associated with recurrent disease following surgery	Tie et al. (42) Pedersen et al. (21)	Retrospective analysis of 230 patients with stage II disease showed ctDNA levels were associated with recurrence-free survival after surgery Prospective study including 12 patients with paired pre- and post-surgery assays showing reduction in mutated ctDNA (BCAT1 and IKZF1) following surgery
ctDNA level changes may be associated with response during systemic treatment for metastatic CRC	Tie et al. (43)	Prospective study of 53 patients with metastatic disease showed association of changes in ctDNA levels with radiographic responses
**ctDNA TO DETECT RESISTANCE TO SYSTEMIC THERAPIES AND GUIDE TREATMENT SELECTION**		
ctDNA levels may be associated with anti-EGFR resistance	Misale et al. (44) Mohan et al. (45) Sclafani et al. (46)	Retrospective analysis of 21 patients showed correlation of ctDNA levels with anti-EGFR response Prospective study of 10 patients with metastatic disease showed correlation of ctDNA levels with anti-EGFR response Retrospective analysis of 97 patients with locally advanced rectal cancer did not show survival benefit with ctDNA detection

## The role of ctDNA in colorectal cancer

### Targeted mutations in ctDNA

A number of common and lesser known biomarkers has been the focus of several studies investigating ctDNA in colorectal cancer. Mutated genes encoding KRAS, BRAF, APC, and p53 are among the more common targeted biomarkers, and each of these has been shown to be involved in the carcinogenesis of colorectal tumors. In 2003, Ryan et al. showed that mutant KRAS2 could be detected in the serum of patients with colorectal cancer prior surgery in 41% of cases; the same KRAS2 mutation was confirmed in 53% of resected tumors, supporting the use of ctDNA as a detection method for mutations reflective of the primary tumor (14). Thierry et al. later showed that multiple KRAS mutations and the BRAF V600E mutation could be reliably detected from ctDNA in patients with metastatic colorectal cancer (15). Detection of APC mutations, like KRAS mutations that are thought to be early changes in the development of colorectal cancer, have also been detected with the use of ctDNA, as well as the detection of P53 mutations, which are thought to be involved in later stage development of colorectal cancer (16).

In addition to these better known mutation targets, a number of other potential ctDNA biomarkers has also been studied. The presence of abnormally methylated septin 9 (mSEPT9) DNA, a GTPase involved in a variety of cellular processes related to carcinogenesis, has been shown to correlate with colon cancer at all stages (17, 18). Toth et al. showed that circulating levels of mSEPT9 were detected in patients with colorectal cancer, but not in patients with precancerous adenomas, providing evidence of its ability to discriminate between malignant and benign tumors (19). Other candidate targets for ctDNA detection have been investigated, including PIK3CA, CDH1, BCAT1, IKZF1, and ALX4, among several others (20–27). While the majority of targets have been aimed at detecting colorectal cancer, some of these new targets, such as ALX4, are being investigated to detect precancerous lesions as well (22). Novel targets continue to be investigated, although their contemporary role in the detection of colorectal cancer remains to be seen.

### ctDNA in early and late colorectal cancer

The clinical utility of ctDNA as a diagnostic and prognostic biomarker varies based on its use in early vs. late colorectal cancer. Ideally, detection of ctDNA mutations would be valuable in identifying precancerous lesions or early cancers when intervention would be most beneficial. Historically, the investigation of ctDNA was performed in colorectal cancer patients with metastatic disease or pooled from a cohort having any stage of disease. Lefebure et al. investigated KRAS and RAS-SF2A mutations in 29 patients with metastatic colorectal cancer, showing that 41% of patients had detectable serum mutations that matched the mutations within the primary tumor (28). In patients where ctDNA mutations were detected, the prognosis was significantly worse as compared to patients without detectable ctDNA mutations. A more recent study by Bachet et al. found a much higher correlation between paired plasma and tumor samples with respect to RAS mutations and a cohort of 412 patients (29). Specifically, the correlation between plasma and tumor mutations was ~70% for patients with colorectal liver metastases. Other studies by Bettegowda et al. and Schmiegel et al. showed that the correlation between plasma ctDNA and tumor mutations was similarly high (~90%) in patients with metastatic colorectal disease (30, 31). This was further validated by a large study of nearly 1,400 patients by Strickler et al. (32). For patients with metastatic disease and detectable (often high) levels of plasma ctDNA, many studies report worse disease-free or overall survival compared to patients without detectable ctDNA (33–35). However, while these more recent studies showed a correlation between ctDNA detection, colorectal metastases, and prognosis, it is unclear whether this information will provide superior outcomes for these patients.

In contrast to metastatic disease, a number of studies have investigated whether ctDNA can be used in the detection of early colorectal disease. As concluded by the recent joint review by the American Society of Clinical Oncology (ASCO) and the College American Pathologists (CAP), there is little evidence of the clinical validity of ctDNA in early stage disease (36). Part of this tempering message regarding ctDNA stems from the fact that many of the studies investigating ctDNA in the detection of early stage colorectal disease are comprised by a heterogeneous patient population or do not show correlation of ctDNA mutations with those found within the tumor specimens (37). For example, Lecomte et al. investigated mutated KRAS2 in a small cohort of patients where only 29 of 58 patients had either stage I or II colorectal cancer with the inability to detect mutations in 5/29 of these early stage patients (38). In addition, unlike contemporary studies in metastatic patients, there was a relatively low correlation between detection of KRAS2 mutation in plasma with detection of the same mutation with in the tumor of 45% (29). In a similar study by Lin et al., the sensitivity of ctDNA mutations was relatively low in early stage disease, specifically 24% in stage I colorectal cancer and 45% in stage II (20). In addition, it is known that the proportion of circulating mutated genes is quite small compared to the number of normal circulating DNA fragments, making it challenging to detect ctDNA in patients with low disease burdens (39). In a study by Diehl and colleagues, multiple ctDNA assays were tested for a small cohort of patients with metastatic colorectal cancer, and the median percentage of mutant DNA fragments was only 0.18% (range, 0.005–11.7% for the 10th and 90th percentiles) (38).

Lastly, while there is much enthusiasm to develop new screening techniques based on ctDNA for the detection of precursors to colorectal cancer in asymptomatic patients, there have been no studies showing the benefit of ctDNA in this role (36). Small studies have investigated the value of ctDNA when combined with other biomarkers, such as CEA levels. Mead et al. developed a predictive model incorporating ctDNA mutations for multiple targets and the serum CEA level, resulting in a positive predictive value of ~80% for cancer (40). A similar study by Flamini et al. also developed a prognostic algorithm incorporating ctDNA and CEA levels, showing high diagnostic predictive value for patients with early stage cancers (41). These predictive stools, however, have yet to be validated or be used as the standard of care for early detection of colorectal cancer.

### ctDNA to predict prognosis following surgery and during systemic treatment

In addition to the detection of early or late stage colorectal cancer, applications of ctDNA have been investigated in predicting recurrent disease following surgery as well as response to disease during systemic treatment. Tie et al. performed a study of 230 patients with resected stage II colon cancer treated (42). In this study, the authors detected ctDNA in 8% of patients who did not receive adjuvant chemotherapy, in whom the majority (79%) had recurred. This is in contrast to a 10% recurrence rate in patients in whom no ctDNA was detected. Other studies have suggested similar conclusions, albeit in smaller cohorts and in more heterogeneous populations, including patients with stage I to stage III disease (47, 48). However, whether the use of ctDNA following surgery results in a benefit to patients with detectable levels of ctDNA, or whether this detection represents a lead-time bias of recurrent disease, has not been addressed by these studies.

A greater body of literature has described the use of ctDNA in monitoring the response metastatic disease to systemic therapies. Current monitoring techniques include serum CEA measurements and the use of different imaging modalities, the interpretation of which is typically based on the Response Evaluation Criteria In Solid Tumors (RECIST) criteria (49, 50). These methods, however, are hindered by several limitations. CEA levels may be falsely low in patients whose tumors do not secrete release CEA, and there may be several reasons for falsely elevated CEA levels (51). Use of the RECIST criteria can often prove challenging, as there can be inter-observer variation and heterogeneous responses among different sites of metastases within an individual patient. Thus, ctDNA holds some promise as a more effective means to monitor response to systemic treatments, thus functioning as a predictive biomarker (52, 53). However, limited data exist to support this proposed use for ctDNA. A study conducted by Tie et al. prospectively followed 53 patients with metastatic colorectal cancer receiving first-line chemotherapy, the majority of whom had oxaliplatin-based followed by irinotecan-based chemotherapy with or without bevacizumab (42). Although this cohort was relatively small, significant reductions in ctDNA levels were observed after the first cycle of chemotherapy. These reductions, defined by a >10-fold decrease in the baseline ctDNA, were not statistically significantly associated with progression-free survival (14.7 vs. 8.1 months, *p* = 0.27, for patients with significant ctDNA reductions vs. not) (43). Thus, the clinical utility of ctDNA in monitoring response to systemic treatment remains to be established and warrants further study.

### ctDNA to detect resistance to systemic therapies and guide treatment selection

Multiple tumors have developed mechanisms to evade the effects of systemic treatments. This has been shown for colorectal cancers, with much of the research performed in anti-EGFR resistance. Anti-EGFR monoclonal antibodies, including cetuximab and panitumumab, are used to treat patients with metastatic colorectal cancer, but are ineffective in tumors that have mutations in the RAS pathway (EGFR-RAS-RAF-MEK signaling cascade) (54). Thus, measurement of ctDNA for RAS mutations may help identify patients who would be non-responders to this targeted therapy or monitor patients who later develop resistance to anti-EGFR antibodies (55). In fact, many patients will initially respond to anti-EGFR therapy, but later develop resistance, and measurement of related mutations has been shown to correlate with this change (56, 57). Newer agents have also been investigated with ctDNA levels, such as Sym004, which is a combination of two anti-EGFR monoclonal antibodies, futuximab, and modotuximab (58). In a phase II randomized trial comparing Sym004 with standard second and third line chemotherapy in ~250 patients with acquired resistance to anti-EGFR monoclonal antibodies (cetuximab and panitumumab), an up to 30-fold decrease in EGFR ctDNA was detected in some patients. However, this response did not translate into a survival benefit (58).

One study by Mohan et al. detected mutations in KRAS and MET ctDNA in a small cohort of patients who developed resistance to anti-EGFR therapy (45). In a study by Misale et al., analysis of metastases from a small cohort of patients who developed resistance to anti-EGFR therapy showed the emergence of KRAS amplification or other KRAS mutations in 60% of cases. Interestingly, the KRAS mutant ctDNA were detectable in the blood of anti-EGFR treated patients as early as 10 months before radiographic disease progression (44). Additional studies by this group also reported the detection of ctDNA mutations to NRAS prior to disease progression while on anti-EGFR therapy, and showed using a mouse xenotransplant model that tumor from a colorectal cancer patient who had initially responded and then relapsed while on anti-EGFR therapy significantly responded to combination treatment with both anti-EGFR and anti-MEK inhibition (59). On the contrary, a recent retrospective study by Sclafani et al. analyzed KRAS and BRAF ctDNA in a cohort of about 100 patients with locally advanced CRC, and showed that there was no difference in outcome between patients with or without detectable ctDNA (46).

Taken together, it is still unclear whether ctDNA may lead to improved individualized care for patients who develop resistance to current targeted treatments, such as anti-EGFR therapy, and guide management for the selection new therapies or combination therapies. However, it is important to stress that these findings are based on very small cohorts of patients and should be further evaluated in prospective studies in order to better establish the benefits of using ctDNA to guide these patient treatment decisions.

### Practical aspects of ctDNA in colorectal cancer

There are several commercially available assays used to obtain, process, and analyze ctDNA from patients. As discussed in the joint review by ASCO and CAP, while there is some consensus on the methodology of ctDNA collection, there are still many questions regarding the optimal processing methods for ctDNA analysis (36, 60). Nuances in ctDNA collection and storage can affect the validity of ctDNA quantification, and there is no current consensus or regulation on the standards for these methods (61). Differences in collection and purification methods have been reported with regard to the quality of extracted ctDNA. For example, Kloten et al. recently reported that silica-based membrane methods improved extraction of long cell free DNA fragments, whereas a magnetic bead system improved extraction of short cell free DNA fragments in serum of cancer patients (62). In general, there are a variety assays for ctDNA, and several specific assays for ctDNA derived from patients with colorectal cancer have been used. Many of these incorporate next generation sequencing in order to detect specific mutations in colorectal cancer as described above (63), while others take a broader approach to ctDNA analysis.

Recognizing that abnormally methylated DNA correlated with the presence of colorectal cancer, He and colleagues developed a polymerase chain reaction (PCR) assay to detect the mutation status of several colorectal cancer selected genes (mSEPT9, ALX, TMEFF2) called the MethyLight assay (64). The sensitivities using these three genes as biomarkers for the detection of colorectal cancer were high both in primary tumor tissue and peripheral blood samples (84 and 81%, respectively, with specificities of 87 and 90%). Later studies have expanded on the number of gene mutations analyzed. Some groups, such as Lin et al., have targeted the identification of hypermethylated genes associated with microsatellite instability (MSI), including D5S345, D2S123, BAT25, BAT26, and D17S250 (65). Detection of three or more of these hypermethylated markers was correlated with metastatic disease and worse disease-free survival. Interestingly, Mouliere et al. developed a multi-gene assay detecting seven KRAS mutations and one BRAF mutation (V600E) that was combined with a type of high resolution scanning microscopy. This imaging technique, known as Atomic Force Microscopy, detects low levels of fragmented DNA in the serum (66). This combined approach yielded a positive predictive value ~90% in the detection of colorectal cancer using a cohort of 124 patients. Recognizing the limitations of ctDNA collection due to the low levels of circulating cell free DNA, other groups have developed enrichment methods in order to increase the detection mutated ctDNA (67–69). Indeed, with the wide breath of techniques available for collecting and analyzing ctDNA, further study is warranted to efficiently compare the reliability, reproducibility, and utility of these methods, in addition to gauging the cost effectiveness among these different assays.

## Conclusion

Over 50 years has passed since the credited discovery circulating cell free DNA, and in the last 15 years much attention and enthusiasm has been given to ctDNA as liquid biopsies to obtain diagnostic and prognostic information for patients with cancer, particularly for patients with colorectal cancer. Advances have been made in the application of ctDNA for (1) detecting early or late stage colorectal cancer, (2) generating predictions of response to systemic therapy, (3) using changes in mutated ctDNA to modify systemic treatments, and (4) utilizing ctDNA to for surveillance of disease recurrence following surgery. Yet many of these studies are limited by their retrospective design and small sample size, and therefore the clinical utility of measuring ctDNA for colorectal cancer has yet to be established. In addition, the high number of different assays available to collect, purify, and analyze ctDNA introduces practical challenges to establishing standard clinical use and utility of these methods. Thus, the fervor over ctDNA has recently been tempered (36, 70, 71). Nonetheless, the advantages of ctDNA are promising, and with novel prospective, collaborative studies, the true benefits of ctDNA in colorectal cancer as well as other malignancies may soon become realized.

## Author contributions

EG drafted the manuscript and performed critical review. SB performed critical review.

### Conflict of interest statement

The authors declare that the research was conducted in the absence of any commercial or financial relationships that could be construed as a potential conflict of interest.

## References

[1] Mandel P Metais P. Les acides nucléiques du plasma sanguin chez l'homme. Comptes Rendus des Seances de la Societe de Biologie et de ses Filiales (1948) 142:241–3. 18875018

[2] Siravegna G Bardelli A. Genotyping cell-free tumor DNA in the blood to detect residual disease and drug resistance. Genome Boil. (2014) 15:449. 10.1186/s13059-014-0449-4 25222559 PMC4281953

[3] Leon SA Shapiro B Sklaroff DM Yaros MJ. Free DNA in the serum of cancer patients and the effect of therapy. Cancer Res. (1977) 37:646–50. 837366

[4] Pantel K Alix-Panabieres C. Circulating tumour cells in cancer patients: challenges and perspectives. Trends Mol Med. (2010) 16:398–406. 10.1016/j.molmed.2010.07.001 20667783

[5] Schwarzenbach H Nishida N Calin GA Pantel K. Clinical relevance of circulating cell-free microRNAs in cancer. Nat Rev Clin Oncol. (2014) 11:145–56. 10.1038/nrclinonc.2014.5 24492836

[6] Alix-Panabieres C Cayrefourcq L Mazard T Maudelonde T Assenat E Assou S. Molecular portrait of metastasis-competent circulating tumor cells in colon cancer reveals the crucial role of genes regulating energy metabolism and DNA repair. Clinical Chem. (2017) 63:700–13. 10.1373/clinchem.2016.263582 28007957

[7] Schwarzenbach H Hoon DS Pantel K. Cell-free nucleic acids as biomarkers in cancer patients. Nat Rev Cancer (2011) 11:426–37. 10.1038/nrc3066 21562580

[8] Garcia-Olmo DC Dominguez C Garcia-Arranz M Anker P Stroun M Garcia-Verdugo JM. Cell-free nucleic acids circulating in the plasma of colorectal cancer patients induce the oncogenic transformation of susceptible cultured cells. Cancer Res. (2010) 70:560–7. 10.1158/0008-5472.CAN-09-3513 20068178

[9] Pantel K Alix-Panabieres C. Real-time liquid biopsy in cancer patients: fact or fiction? Cancer Res. (2013) 73:6384–8. 10.1158/0008-5472.CAN-13-2030 24145355

[10] Lopez A Harada K Mizrak Kaya D Dong X Song S Ajani JA. Liquid biopsies in gastrointestinal malignancies: when is the big day? Expert Rev Anticancer Ther. (2018) 18:19–38. 10.1080/14737140.2018.1403320 29202614

[11] Mangano A Mangano A Lianos GD Cassinotti E Roukos DH Dionigi G. Circulating free DNA in plasma or serum as biomarkers of carcinogenesis in colon cancer. Future Oncol. (2015) 11:1455–8. 10.2217/fon.15.63 25963422

[12] Mouliere F El Messaoudi S Gongora C Guedj AS Robert B Del Rio M. Circulating cell-free DNA from colorectal cancer patients may reveal high KRAS or BRAF mutation load. Transl Oncol. (2013) 6:319–28. 10.1593/tlo.12445 23730412 PMC3660801

[13] Mouliere F Robert B Arnau Peyrotte E Del Rio M Ychou M Molina F. High fragmentation characterizes tumour-derived circulating DNA. PLoS ONE (2011) 6:e23418. 10.1371/journal.pone.0023418 21909401 PMC3167805

[14] Ryan BM Lefort F McManus R Daly J Keeling PW Weir DG. A prospective study of circulating mutant KRAS2 in the serum of patients with colorectal neoplasia: strong prognostic indicator in postoperative follow up. Gut (2003) 52:101–8. 10.1136/gut.52.1.101 12477769 PMC1773535

[15] Thierry AR Mouliere F El Messaoudi S Mollevi C Lopez-Crapez E Rolet F. Clinical validation of the detection of KRAS and BRAF mutations from circulating tumor DNA. Nat Med. (2014) 20:430–5. 10.1038/nm.3511 24658074

[16] Wang JY Hsieh JS Chang MY Huang TJ Chen FM Cheng TL. Molecular detection of APC, K- ras, and p53 mutations in the serum of colorectal cancer patients as circulating biomarkers. World J Surg. (2004) 28:721–6. 10.1007/s00268-004-7366-8 15185002

[17] deVos T Tetzner R Model F Weiss G Schuster M Distler J. Circulating methylated SEPT9 DNA in plasma is a biomarker for colorectal cancer. Clin Chem. (2009) 55:1337–46. 10.1373/clinchem.2008.115808 19406918

[18] Grutzmann R Molnar B Pilarsky C Habermann JK Schlag PM Saeger HD. Sensitive detection of colorectal cancer in peripheral blood by septin 9 DNA methylation assay. PLoS ONE (2008) 3:e3759. 10.1371/journal.pone.0003759 19018278 PMC2582436

[19] Toth K Wasserkort R Sipos F Kalmar A Wichmann B Leiszter K. Detection of methylated septin 9 in tissue and plasma of colorectal patients with neoplasia and the relationship to the amount of circulating cell-free DNA. PLoS ONE (2014) 9:e115415. 10.1371/journal.pone.0115415 25526039 PMC4272286

[20] Lin JK Lin PC Lin CH Jiang JK Yang SH Liang WY. Clinical relevance of alterations in quantity and quality of plasma DNA in colorectal cancer patients: based on the mutation spectra detected in primary tumors. Ann Surg Oncol. (2014) 21(Suppl. 4):S680–6. 10.1245/s10434-014-3804-5 24841357

[21] Pedersen SK Symonds EL Baker RT Murray DH McEvoy A Van Doorn SC. Evaluation of an assay for methylated BCAT1 and IKZF1 in plasma for detection of colorectal neoplasia. BMC Cancer (2015) 15:654. 10.1186/s12885-015-1674-2 26445409 PMC4596413

[22] Tanzer M Balluff B Distler J Hale K Leodolter A Rocken C. Performance of epigenetic markers SEPT9 and ALX4 in plasma for detection of colorectal precancerous lesions. PLoS ONE (2010) 5:e9061. 10.1371/journal.pone.0009061 20140221 PMC2816214

[23] Bhangu JS Taghizadeh H Braunschmid T Bachleitner-Hofmann T Mannhalter C. Circulating cell-free DNA in plasma of colorectal cancer patients - a potential biomarker for tumor burden. Surg Oncol. (2017) 26:395–401. 10.1016/j.suronc.2017.08.001 29113658

[24] Xue G Lu CJ Pan SJ Zhang YL Miao H Shan S. DNA hypomethylation of CBS promoter induced by folate deficiency is a potential noninvasive circulating biomarker for colorectal adenocarcinomas. Oncotarget (2017) 8:51387–401. 10.18632/oncotarget.17988 28881655 PMC5584256

[25] Boni L Cassinotti E Canziani M Dionigi G Rovera F Dionigi R. Free circulating DNA as possible tumour marker in colorectal cancer. Surg Oncol. (2007) 16(Suppl. 1):S29–31. 10.1016/j.suronc.2007.10.004 18024018

[26] Kasi PM. Mutational burden on circulating cell-free tumor-DNA testing as a surrogate marker of mismatch repair deficiency or microsatellite instability in patients with colorectal cancers. J Gastrointest Oncol. (2017) 8:747–8. 10.21037/jgo.2017.06.05 28890826 PMC5582036

[27] Umetani N Kim J Hiramatsu S Reber HA Hines OJ Bilchik AJ. Increased integrity of free circulating DNA in sera of patients with colorectal or periampullary cancer: direct quantitative PCR for ALU repeats. Clin Chem. (2006) 52:1062–9. 10.1373/clinchem.2006.068577 16723681

[28] Lefebure B Charbonnier F Di Fiore F Tuech JJ Le Pessot F Michot F. Prognostic value of circulating mutant DNA in unresectable metastatic colorectal cancer. Ann Surg (2010) 251:275–80. 10.1097/SLA.0b013e3181c35c87 20010083

[29] Bachet JB Bouche O Taieb J Dubreuil O Garcia ML Meurisse A. RAS mutation analysis in circulating tumor DNA from patients with metastatic colorectal cancer: the AGEO RASANC prospective multicenter study. Ann Oncol. (2018). 29:1211–9. 10.1093/annonc/mdy061 29438522

[30] Bettegowda C Sausen M Leary RJ Kinde I Wang Y Agrawal N. Detection of circulating tumor DNA in early- and late-stage human malignancies. Sci Transl Med. (2014) 6:224ra224. 10.1126/scitranslmed.3007094 24553385 PMC4017867

[31] Schmiegel W Scott RJ Dooley S Lewis W Meldrum CJ Pockney P. Blood-based detection of RAS mutations to guide anti-EGFR therapy in colorectal cancer patients: concordance of results from circulating tumor DNA and tissue-based RAS testing. Mol Oncol. (2017) 11:208–19. 10.1002/1878-0261.12023 28106345 PMC5527457

[32] Strickler JH Loree JM Ahronian LG Parikh AR Niedzwiecki D Pereira AAL. Genomic landscape of cell-free DNA in patients with colorectal cancer. Cancer Discov. (2018) 8:164–73. 10.1158/2159-8290.CD-17-1009 29196463 PMC5809260

[33] Fan G Zhang K Yang X Ding J Wang Z Li J. Prognostic value of circulating tumor DNA in patients with colon cancer: systematic review. PLoS ONE (2017) 12:e0171991. 10.1371/journal.pone.0171991 28187169 PMC5302475

[34] Spindler KL Pallisgaard N Andersen RF Brandslund I Jakobsen A. Circulating free DNA as biomarker and source for mutation detection in metastatic colorectal cancer. PLoS ONE (2015) 10:e0108247. 10.1371/journal.pone.0108247 25875772 PMC4395277

[35] Trevisiol C Di Fabio F Nascimbeni R Peloso L Salbe C Ferruzzi E. Prognostic value of circulating KRAS2 gene mutations in colorectal cancer with distant metastases. Int J Biol Mark. (2006) 21:223–28. 10.1177/172460080602100405 17177160

[36] Merker JD Oxnard GR Compton C Diehn M Hurley P Lazar AJ. Circulating tumor DNA analysis in patients with cancer: American Society of Clinical Oncology and College of American pathologists joint review. J Clin Oncol. (2018) 36:1631–41. 10.1200/JCO.2017.76.8671 29504847

[37] Vietsch EE Graham GT McCutcheon JN Javaid A Giaccone G Marshall JL. Circulating cell-free DNA mutation patterns in early and late stage colon and pancreatic cancer. Cancer Genet. (2017) 218–219:39–50. 10.1016/j.cancergen.2017.08.006 29153095 PMC5726797

[38] Lecomte T Berger A Zinzindohoue F Micard S Landi B Blons H. Detection of free-circulating tumor-associated DNA in plasma of colorectal cancer patients and its association with prognosis. Int J Cancer (2002) 100:542–8. 10.1002/ijc.10526 12124803

[39] Diehl F Schmidt K Choti MA Romans K Goodman S Li M. Circulating mutant DNA to assess tumor dynamics. Nat Med. (2008) 14:985–90. 10.1038/nm.1789 18670422 PMC2820391

[40] Mead R Duku M Bhandari P Cree IA. Circulating tumour markers can define patients with normal colons, benign polyps, and cancers. Br J Cancer (2011) 105:239–45. 10.1038/bjc.2011.230 21712823 PMC3142810

[41] Flamini E Mercatali L Nanni O Calistri D Nunziatini R Zoli W. Free DNA and carcinoembryonic antigen serum levels: an important combination for diagnosis of colorectal cancer. Clin Cancer Res. (2006) 12:6985–88. 10.1158/1078-0432.CCR-06-1931 17145818

[42] Tie J Wang Y Tomasetti C Li L Springer S Kinde I. Circulating tumor DNA analysis detects minimal residual disease and predicts recurrence in patients with stage II colon cancer. Sci Transl Med. (2016) 8:346ra392. 10.1126/scitranslmed.aaf6219 27384348 PMC5346159

[43] Tie J Kinde I Wang Y Wong HL Roebert J Christie M. Circulating tumor DNA as an early marker of therapeutic response in patients with metastatic colorectal cancer. Ann Oncol. (2015) 26:1715–22. 10.1093/annonc/mdv177 25851626 PMC4511218

[44] Misale S Yaeger R Hobor S Scala E Janakiraman M Liska D. Emergence of KRAS mutations and acquired resistance to anti-EGFR therapy in colorectal cancer. Nature (2012) 486:532–6. 10.1038/nature11156 22722830 PMC3927413

[45] Mohan S Heitzer E Ulz P Lafer I Lax S Auer M. Changes in colorectal carcinoma genomes under anti-EGFR therapy identified by whole-genome plasma DNA sequencing. PLoS Genet. (2014) 10:e1004271. 10.1371/journal.pgen.1004271 24676216 PMC3967949

[46] Sclafani F Chau I Cunningham D Hahne JC Vlachogiannis G Eltahir Z. KRAS and BRAF mutations in circulating tumour DNA from locally advanced rectal cancer. Sci Rep. (2018) 8:1445. 10.1038/s41598-018-19212-5 29362371 PMC5780472

[47] Reinert T Scholer LV Thomsen R Tobiasen H Vang S Nordentoft I. Analysis of circulating tumour DNA to monitor disease burden following colorectal cancer surgery. Gut (2016) 65:625–34. 10.1136/gutjnl-2014-308859 25654990

[48] Frattini M Gallino G Signoroni S Balestra D Battaglia L Sozzi G. Quantitative analysis of plasma DNA in colorectal cancer patients: a novel prognostic tool. Ann NY Acad Sci. (2006) 1075:185–90. 10.1196/annals.1368.025 17108210

[49] Vukobrat-Bijedic Z Husic-Selimovic A Sofic A Bijedic N Bjelogrlic I Gogov B. Cancer antigens (CEA and CA 19-9) as markers of advanced stage of colorectal carcinoma. Med Arch. (2013) 67:397–401. 10.5455/medarh.2013.67.397-401 25568506 PMC4272469

[50] Solinas C Porcu M Hlavata Z De Silva P Puzzoni M Willard-Gallo K. Critical features and challenges associated with imaging in patients undergoing cancer immunotherapy. Crit Rev Oncol hematol. (2017) 120:13–21. 10.1016/j.critrevonc.2017.09.017 29198327

[51] Wahlby L. [Carcinoembryonic antigen, CEA, in colorectal cancer. An insensitive marker which may be excluded from follow-ups]. Lakartidningen (1997) 94:1716–8. 9182180

[52] Berger AW Schwerdel D Welz H Marienfeld R Schmidt SA Kleger A. Treatment monitoring in metastatic colorectal cancer patients by quantification and KRAS genotyping of circulating cell-free DNA. PLoS ONE (2017) 12:e0174308. 10.1371/journal.pone.0174308 28328955 PMC5362218

[53] Siravegna G Bardelli A. Blood circulating tumor DNA for non-invasive genotyping of colon cancer patients. Mol Oncol. (2016) 10:475–80. 10.1016/j.molonc.2015.12.005 26774880 PMC5528968

[54] Karapetis CS Khambata-Ford S Jonker DJ O'Callaghan CJ Tu D Tebbutt NC. K-ras mutations and benefit from cetuximab in advanced colorectal cancer. N Engl J Med. (2008) 359:1757–65. 10.1056/NEJMoa0804385 18946061

[55] Van Emburgh BO Sartore-Bianchi A Di Nicolantonio F Siena S Bardelli A. Acquired resistance to EGFR-targeted therapies in colorectal cancer. Mol Oncol. (2014) 8:1084–94. 10.1016/j.molonc.2014.05.003 24913799 PMC5528615

[56] Misale S Di Nicolantonio F Sartore-Bianchi A Siena S Bardelli A. Resistance to anti-EGFR therapy in colorectal cancer: from heterogeneity to convergent evolution. Cancer Discov. (2014) 4:1269–80. 10.1158/2159-8290.CD-14-0462 25293556

[57] Siravegna G Mussolin B Buscarino M Corti G Cassingena A Crisafulli G Clonal evolution and resistance to EGFR blockade in the blood of colorectal cancer patients. Nat Med. (2015) 21:827 10.1038/nm0715-827b 26151329

[58] Montagut C Argiles G Ciardiello F Poulsen TT Dienstmann R Kragh M. Efficacy of Sym004 in patients with metastatic colorectal cancer with acquired resistance to anti-EGFR therapy and molecularly selected by circulating tumor DNA analyses: a phase 2 randomized clinical trial. JAMA Oncol. (2018) 4:e175245. 10.1001/jamaoncol.2017.5245 29423521 PMC5885274

[59] Misale S Arena S Lamba S Siravegna G Lallo A Hobor S. Blockade of EGFR and MEK intercepts heterogeneous mechanisms of acquired resistance to anti-EGFR therapies in colorectal cancer. Sci Transl Med. (2014) 6:224ra226. 10.1126/scitranslmed.3007947 24553387

[60] ctDNA Analysis for cancer? Not so fast. Cancer Discov. (2018) 8:526 10.1158/2159-8290.CD-NB2018-033 29572234

[61] El Messaoudi S Rolet F Mouliere F Thierry AR. Circulating cell free DNA: preanalytical considerations. Clin Chim Acta Int J Clin Chem. (2013) 424:222–30. 10.1016/j.cca.2013.05.022 23727028

[62] Kloten V Ruchel N Bruchle NO Gasthaus J Freudenmacher N Steib F. Liquid biopsy in colon cancer: comparison of different circulating DNA extraction systems following absolute quantification of KRAS mutations using Intplex allele-specific PCR. Oncotarget (2017) 8:86253–63. 10.18632/oncotarget.21134 29156792 PMC5689682

[63] Rachiglio AM Esposito Abate R Sacco A Pasquale R Fenizia F Lambiase M. Limits and potential of targeted sequencing analysis of liquid biopsy in patients with lung and colon carcinoma. Oncotarget (2016) 7:66595–605. 10.18632/oncotarget.10704 27448974 PMC5341823

[64] He Q Chen HY Bai EQ Luo YX Fu RJ He YS. Development of a multiplex MethyLight assay for the detection of multigene methylation in human colorectal cancer. Cancer Genet Cytogenet. (2010) 202:1–10. 10.1016/j.cancergencyto.2010.05.018 20804913

[65] Lin PC Lin JK Lin CH Lin HH Yang SH Jiang JK. Clinical relevance of plasma DNA methylation in colorectal cancer patients identified by using a genome-wide high-resolution array. Ann Surg Oncol. (2015) 22(Suppl. 3):S1419–27. 10.1245/s10434-014-4277-2 25472652

[66] Mouliere F El Messaoudi S Pang D Dritschilo A Thierry AR. Multi-marker analysis of circulating cell-free DNA toward personalized medicine for colorectal cancer. Mol Oncol. (2014) 8:927–41. 10.1016/j.molonc.2014.02.005 24698732 PMC5528519

[67] Sefrioui D Mauger F Leclere L Beaussire L Di Fiore F Deleuze JF. Comparison of the quantification of KRAS mutations by digital PCR and E-ice-COLD-PCR in circulating-cell-free DNA from metastatic colorectal cancer patients. Clin Chim Acta (2017) 465:1–4. 10.1016/j.cca.2016.12.004 27940131

[68] How Kit A Mazaleyrat N Daunay A Nielsen HM Terris B Tost J. Sensitive detection of KRAS mutations using enhanced-ice-COLD-PCR mutation enrichment and direct sequence identification. Hum Mutat. (2013) 34:1568–80. 10.1002/humu.22427 24038839

[69] Carotenuto P Roma C Cozzolino S Fenizia F Rachiglio AM Tatangelo F. Detection of KRAS mutations in colorectal cancer with Fast COLD-PCR. Int J Oncol. (2012) 40:378–84. 10.3892/ijo.2011.1221 21971641

[70] Montagut C Tsui DW Diaz LA Jr. Detection of somatic RAS mutations in circulating tumor DNA from metastatic colorectal cancer patients: are we ready for clinical use? Ann Oncol. (2018) 29:1083–4. 10.1093/annonc/mdy091 29554200

[71] Vivancos A Elez E Salazar R. Circulating cell-free DNA as predictor of treatment failure after neoadjuvant chemoradiotherapy before surgery in patients with locally advanced rectal cancer: is it ready for primetime? Ann Oncol. (2018) 29:532–4. 10.1093/annonc/mdy043 29401228

